# VISION 2020 INDIA's annual conference

**Published:** 2018-07-31

**The key outcomes of the VISION 2020 INDIA's annual conference this year was the submission of six recommendations on improving the Health Management Information System (HMIS) to the National Programme for Control of Blindness (NPCB), the nodal body in charge of blindness programmes in India.**

**Figure:**
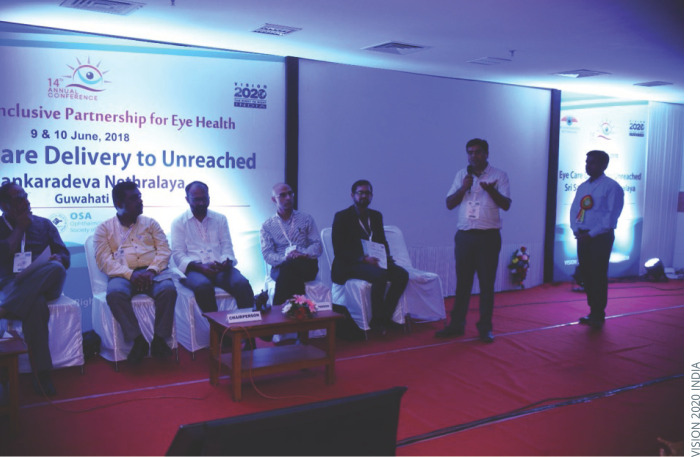
A session in progress at the conference. INDIA

VISION 2020 INDIA's annual conference titled ‘advocacy and inclusive partnership for eye health’ was hosted by Sri Sankaradeva Nethralaya, Guwahati, Assam on 9 and 10 June this year. This time the conference was held in the north east of India, a region where delivering eye health is a major challenge. The states of Assam and Arunachal Pradesh, for example, have the highest and the second highest prevalence of blindness in the country respectively. More than 500 delegates attended the conference, which had a mix of senior officials from both the central and state governments, heads of organisations, ophthalmologists, programme managers, optometrists and mid-level ophthalmic personnel.

**Figure:**
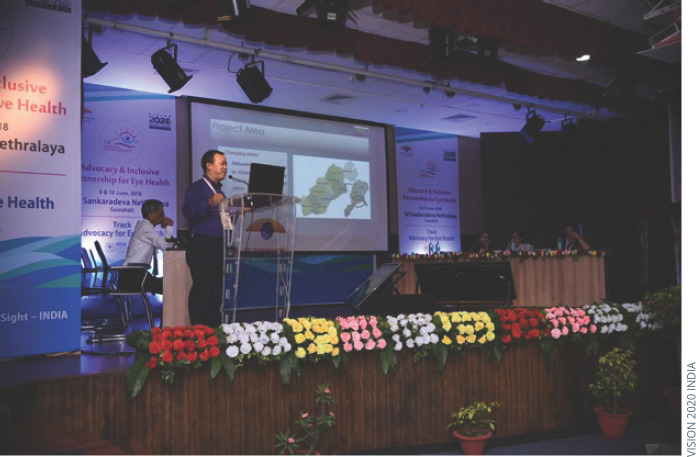
Dr Taba Khanna, State Programme Officer, Arunachal Pradesh, NPCB presenting challenges in northeast region. INDIA

One of the key outcomes of the conference was the submission of six recommendations on improving the Health Management Information System (HMIS) to the National Programme for Control of Blindness (NPCB), the nodal body in charge of blindness programmes in India. A robust and glitch-free HMIS system is vital in the Indian scenario, where several not-for-profit eye care organisations perform cataract surgeries under the grant–in– aid programmes of the government and HMIS is the key monitoring tool for grant reimbursements. “Currently NPCB is in the process of revamping the HMIS and recommendations from organisations based on their experiences will help in building a strong HMIS,” says Mr Phanindra Babu Nukella, CEO, VISION 2020 INDIA.

A session on ‘challenges in delivering eye health in northeast region’ highlighted some of the success stories from the region and also recommended improvements. The session was chaired by Dr Promila Gupta, Director General Health Services, Ministry of Health and Family Welfare, Government of India and co-chaired by Dr Dipali Deka, RIO, Assam.

His Excellency, Shri Jagdish Mukhi, Governor of Assam, inaugurated the conference. In his speech, he hoped that the deliberations can be the “key to solve the issue” of Assam having the highest blindness prevalence rate in the country.

Over the years, the annual conference has metamorphosed into a national conference for community ophthalmology. “This conference is an unique opportunity for learning which is generally not available in the more commonly held CMEs that are more clinical in nature,” said Dr T P Das, President, VISION 2020 INDIA. The conference is uniquely positioned to discuss and promote delivery aspects of community eye care and has something useful for all departments of a hospital.

This year's conference included four tracks: advocacy for eye health; eye care delivery to the unreached; improving patient outcomes in cataract surgery and; skill enhancement for optometrists and ophthalmic assistants. 20 sessions enabled debates, discussions, experience and knowledge sharing from organisations across the country.

